# Relating Acute Lesion Loads to Chronic Outcome in Ischemic Stroke–An Exploratory Comparison of Mismatch Patterns and Predictive Modeling

**DOI:** 10.3389/fneur.2018.00737

**Published:** 2018-09-11

**Authors:** Simon Habegger, Roland Wiest, Bruno J. Weder, Pasquale Mordasini, Jan Gralla, Levin Häni, Simon Jung, Mauricio Reyes, Richard McKinley

**Affiliations:** ^1^Support Center for Advanced Neuroimaging, Institute for Diagnostic and Interventional Neuroradiology, Inselspital, University of Bern, Bern, Switzerland; ^2^Department of Neurosurgery, Inselspital, University of Bern, Bern, Switzerland; ^3^Department of Neurology, Inselspital, University of Bern, Bern, Switzerland; ^4^Neurovascular Imaging Research Core, Department of Neurology, University of California, Los Angeles, Los Angeles, CA, United States; ^5^Institute for Surgical Technology and Biomechanics, University of Bern, Bern, Switzerland

**Keywords:** stroke recovery, lesion load, correlation, FASTER, atlas-based regional image analysis

## Abstract

**Objectives:** To investigate the relationship between imaging features derived from lesion loads and 3 month clinical assessments in ischemic stroke patients. To support clinically implementable predictive modeling with information from lesion-load features.

**Methods:** A retrospective cohort of ischemic stroke patients was studied. The dataset was dichotomized based on revascularization treatment outcome (TICI score). Three lesion delineations were derived from magnetic resonance imaging in each group: two clinically implementable (threshold based and fully automatic prediction) and 90-day follow-up as final groundtruth. Lesion load imaging features were created through overlay of the lesion delineations on a histological brain atlas, and were correlated with the clinical assessment (NIHSS). Significance of the correlations was assessed by constructing confidence intervals using bootstrap sampling.

**Results:** Overall, high correlations between lesion loads and clinical score were observed (up to 0.859). Delineations derived from acute imaging yielded on average somewhat lower correlations than delineations derived from 90-day follow-up imaging. Correlations suggest that both total lesion volume and corticospinal tract lesion load are associated with functional outcome, and in addition highlight other potential areas associated with poor clinical outcome, including the primary somatosensory cortex BA3a. Fully automatic prediction was comparable to ADC threshold-based delineation on the successfully treated cohort and superior to the Tmax threshold-based delineation in the unsuccessfully treated cohort.

**Conclusions:** The confirmation of established predictors for stroke outcome (e.g., corticospinal tract integrity and total lesion volume) gives support to the proposed methodology—relating acute lesion loads to 3 month outcome assessments by way of correlation. Furthermore, the preliminary results indicate an association of further brain regions and structures with three month NIHSS outcome assessments. Hence, prediction models might observe an increased accuracy when incorporating regional (instead of global) lesion loads. Also, the results lend support to the clinical utilization of the automatically predicted volumes from FASTER, rather than the simpler DWI and PWI lesion delineations.

## Background

In 2013, 18.3 million ischemic stroke survivors were reported world-wide. The incidence of ischemic stroke in the same year was stated to be 6.9 million and the disease claimed 3.3 million lives worldwide (1). The global burden of ischemic stroke has increased with respect to incidence (37%), number of death (21%), and DALYs lost (18%) over the last two decades (2). Ischemic stroke has an enormous individual, socioenvironmental and economic impact; improvements in stroke treatment and rehabilitation may therefore be of great societal interest.

An accurate assessment of likely neurological deficits after an acute stroke is important for various reasons, including setting attainable treatment goals, correctly and accurately informing patients and relatives, planning facility discharge, and assessing impact on daily living (3). Additionally, if this assessment is available at the acute stage, it may be possible to better stratify patients who are eligible for mechanical thrombectomy. Total lesion volume has been found to be an independent 90 days predictor of neurological outcome (4), and lesion topography is related to recovery and outcome prognosis (5–8). The Alberta Stroke Program Early Computed Tomography Score (ASPECTS) was created to quantify ischemic changes in ten regions along the middle cerebral artery (9). It is linked to 3 month functional outcome as measured by mRS (10) and values > 6 were found to be predictive for functional independence at 3 months and 1 year post-stroke (11). Diffusion-weighted imaging (DWI) provides an early depiction of size and location of an ischemic lesion. DWI lesion volume is an independent predictor of Barthel Index (BI) quantified outcome (12) and the power of prediction models may be increased by incorporating it as a feature (13).

Existing models predicting clinical outcome from acute imaging have taken into account load on the corticospinal tract (14–16) and lesion location (17, 18). In order for such a model to be useful for treatment selection, the model must operate within the acute time-frame. Images must be processed with little or no human interaction, meaning that manual lesion delineation is impossible. Systems providing fast automated definitions of tissue-at-risk would, on the other hand, be feasible in the acute setting. This paper investigates lesion-load features based on three different lesion delineations to demonstrate the plausibility of automatically linking lesion loads to clinical outcome. First, we analyze the correlation between observed lesion loads and outcome, as given by a manual segmentation of 90 day follow-up imaging. Second, we analyze the correlation between predicted lesion load at the acute stage and outcome: in addition to the standard threshold-based concepts of core and penumbra, we derive predicted lesion loads from the prediction maps of a previously proposed in-house developed software (19). We perform an exploratory analysis of the plausibility of automatically linking lesion loads to clinical outcome, using a small retrospective cohort to relate a large number of imaging features to outcome. We conjecture that (i) a significant relationship exists between lesion load features and clinical assessments, where both are assessed in the chronic phase of the disease, (ii) that (i) is still valid if the features are extracted from an automatic lesion delineation at the acute stage, and, hence, that the method is clinically applicable, and (iii) that the lesion prediction maps from a previously proposed in-house developed software (19) are superior to a simple threshold-derived lesion delineation (i.e., Tmax > 6 s) for finding a relationship between lesion loads at the acute phase and clinical outcome assessments.

## Materials and methods

### Study ethics

The study is based on data from the Bernese stroke registry, a prospectively collected database approved by the Kantonale Ethikkomission Bern, some aspects of which have been reported previously (20–23). All patients were treated for an acute ischemic stroke at the University Hospital of Bern between 2005 and 2013. The study was performed according to the ethical guidelines of the Canton of Bern with approval of our institutional review board (Kantonale Ethikkomission Bern).

### Inclusion criteria

Patients were included in this analysis if: (i) a diagnosis of ischemic stroke was established by MR imaging with an identifiable lesion on DWI and perfusion imaging, (ii) a proximal occlusion of the middle cerebral artery (M1 or M2 segment) was documented on digital subtraction angiography, (iii) endovascular therapy was attempted, either by intra-arterial thrombolysis (before 2010) or by mechanical thrombectomy (since 2010), (iv) pre-treatment MRI was performed with sufficient quality (i.e., no motion artifacts), (v) the imaging data were recorded completely into the picture archiving and communication system, (vi) the patients had a minimum age of 18 years at the time of stroke. Patients were excluded if they received only purely diagnostic angiography. Patients with a stenosis or occlusion of the carotid artery were excluded as well. Revascularization success was stratified retrospectively according to the TICI score by two examiners blinded for clinical data (24). Stroke severity for these patients was assessed at admission according to the National Institutes of Health Stroke Scale (NIHSS) scale. We aimed to identify all patients with a 3-month axial T2-weighted follow-up image in order to define the final extent of infarction. The inclusion/exclusion criteria did not depend on lesion location, nor was the data selected according to predetermined impairments.

### Clinical assessment

The degree of recovery was determined with standard scores (NIHSS and mRS) that are routinely available in clinical stroke registries.

### Dataset splitting

After endovascular therapy, success of the intervention can be determined via the TICI score (24). In the study at hand, patients were dichotomized according to endovascular therapy outcome into successful and unsuccessful revascularization. Successful revascularization was ascribed to patients with a TICI score 2b-3 whereas the unsuccessful revascularization was assigned to TICI scores 0-2a.

### Pipeline

**Figure 1:**
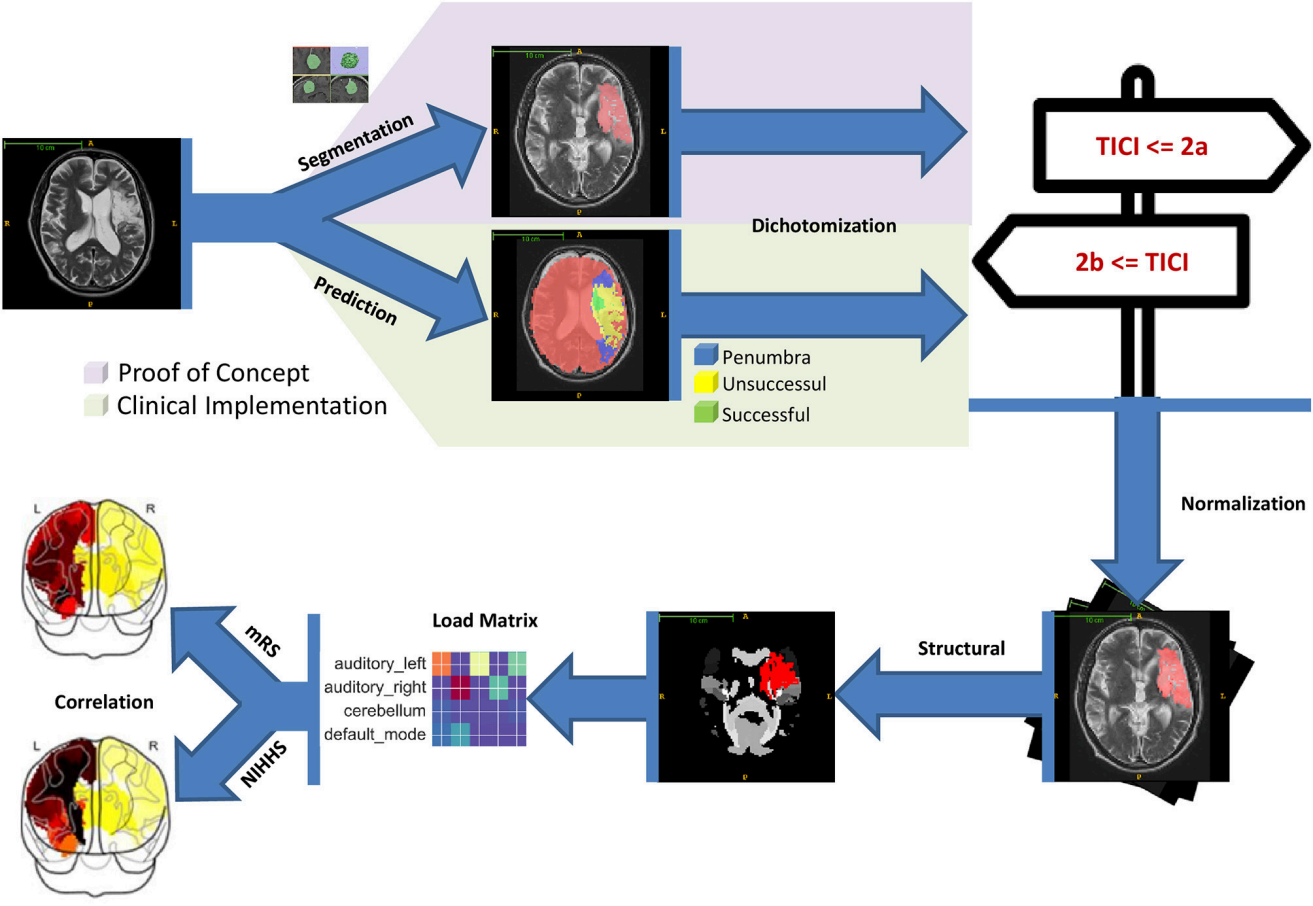
Starting with the MRI imaging data lesion delineations were generated either manually or automatically. The former was used for proof of concept and the latter to show the proposed clinical implementation. The lesion delineations were then dichotomized into successful and unsuccessful revascularization groups according to the patient's TICI scores. Image normalization was performed in a next step to make them conform to MNI152 space. With that, the lesion delineations were superimposed onto the structural atlas and lesion loads for every region computed. Finally, the lesion loads were correlated with the 3 month mRS and NIHSS scores.

The processing pipeline used in this paper is depicted in Figure 1. The individual steps are briefly discussed in the following sections.

### Image acquisition

Imaging data were acquired on either a 1.5T (Siemens Magnetom Avanto) or 3T MRI system (Siemens Magnetom Verio). Patients received whole brain DWI, (24 slices, thickness 5 mm, repetition time 3,200 ms, echo time 87 ms, number of averages 3, matrix 256 × 256, flip angle 90) yielding images for b values of 0 s/mm^2^ and 1,000 s/mm^2^ as well as ADC maps that were calculated automatically. Standard dynamic susceptibility contrast-enhanced perfusion MRI (gradient-echo echoplanar imaging sequence, repetition time 1,410 ms, echo time 30 ms, field of view 230 × 230 mm, voxel size: 1.8 × 1.8 × 5.0 mm, slice thickness 5.0 mm, 19 slices, 80 acquisitions, flip angle 90) was acquired. Images were acquired during the first pass of a standard bolus of 0.1 mmol/kg gadobutrol (Gadovist, Bayer Healthcare). Contrast medium was injected at a rate of 5 ml/s followed by a 20 ml bolus of saline at a rate of 5 ml/s. In addition, an axial T2-weighted turbo-spin echo sequence (TR 3760–4100 ms, TE 85–100 ms, flip angle 150) and contrast-enhanced T1-weighted sequence [1.5T system: spin-echo sequence (TR 663 ms, TE 17 ms, flip angle 90), 3T system: gradient-echo sequence (TR 250 ms, TE 2.67 ms, flip angle 70)], a time-of-flight angiography and a first pass Gd-MRA were acquired, with T2-weighted imaging and TOF angiography performed before contrast injection.

#### Pre-processing

The main pre-processing step in this work was image normalization to warp the utilized images into the MNI152 space with a 2 × 2 × 2 mm resolution. This was done in Matlab (MATLAB R2014a, The MathWorks, Inc., Natick, Massachusetts, United States) with SPM12 (Wellcome Trust Centre for Neuroimaging, University College London).

#### Lesion delineation

We compared four lesion maps: expert manual segmentation of 90 day T2 MRI, threshold-based manual segmentation of acute ADC, automated threshold-based segmentation of Tmax imaging, and machine-learning-based prediction of chronic lesion load from acute imaging (using our in-house software tool, FASTER).

#### FASTER

FASTER (19) is a recently proposed stroke lesion estimation method. Given acute stroke imaging data, FASTER produces two predicted lesion maps, representing successful and unsuccessful revascularization. The required inputs to FASTER consist of diffusion-weighted, T2 and T1w (contrast enhanced) sequences and dynamic susceptibility contrast perfusion. FASTER provides a threshold-independent estimation by using two machine-learning models trained on cases with a TICI of 3 and 0, respectively. The fully automated software makes stroke outcome prediction feasible in any clinical setting, provided the necessary imaging data is accessible. Using FASTER, a prediction of tissue damage in the case of successful revascularization was calculated for the patients with TICI 0-2a, and a prediction of tissue damage in the case of unsuccessful revascularization was calculated for the patients with TICI 2b-3.

#### Follow-up segmentation

Manual segmentation was performed on the T2-weighted 3 month follow-up images. Manual regions of interest were drawn to the maximal extent of the final infarction, including areas with hemorrhagic transformation, but excluding regions already hyperintense on acute T2 imaging. The boundaries of the infarctions were manually delineated for every single transversal slice. The 3 month follow-up lesion was chosen as the definition of final infarction, rather than the lesion in the early acute phase of lesion evolution, since apparent lesion size in the early acute phase is known to overestimate final lesion volume (25). T2 was chosen as the modality for identifying the final lesion, since it was more widely available than a FLAIR follow-up image in the retrospective data used.

#### Core and Tmax > 6 s delineations

The infarct core was manually segmented based on a threshold of ADC < 600 ^*^ 10^−6^ mm^2^/s (26). The perfusion deficit was computed as a pre-processing step of FASTER, using a threshold of Tmax > 6 s (27).

### Lesion load features

**Figure 2:**
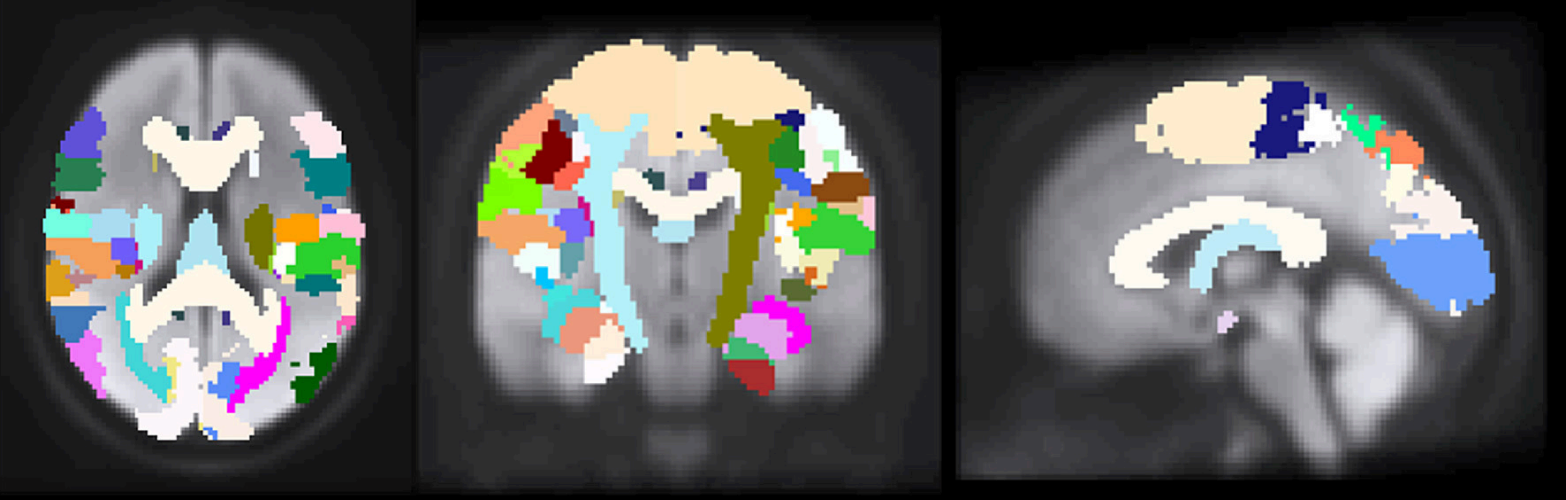
The images depict axial, coronal and sagittal slices of a normalized brain with the overlapped Juelich histological atlas. Both gray and white matter structures can be seen.

Previous studies that investigated the relationship between imaging biomarkers and clinical outcome assessments have revealed the importance of white-matter structures (15, 16, 28, 29). Therefore, it was important to choose an atlas for feature definition that identifies white-matter structures, especially, left and right corticospinal tract. To this end the Juelich histological atlas (30–32) from FSL 5.0 was selected, which encompasses 29 white-matter and 92 gray-matter regions (Figure 2).

Following normalization, the lesion segmentations and predictions were overlaid onto the Juelich structural atlas. After that, a load percentage was computed for every structural region of interest. The lesion load denotes the percentage of the region that was affected by the lesion.

### Correlation analysis

Lesion loads were created on a per-patient level for every atlas defined region based on the lesion segmentation—i.e., 121 lesion loads per patient. The loads designate the percentage of the respective region affected by the lesion.

The correlation between the 3 month clinical assessments and the lesion load of every atlas-defined region was assessed.

The presented work rests on the premise that a linear relationship between continuous lesion loads and continuous 3 month clinical assessment scores exists. As a result, the Pearson correlation coefficient was selected to assess the relationship.

Correlations were also calculated between total volume and outcome.

In a first step, we correlated lesion loads with both 3 month NIHSS and mRS scores. The whole dataset was considered (i.e., no grouping into successful and unsuccessful) and the lesion loads were based on the 3 month follow-up segmentation. The subsequent analysis was then carried out with only the superior clinical assessment that emerged from this analysis. In a second step, we evaluated the various lesion delineations by correlating the respective lesion loads with the superior outcome measure from the first step (i.e., 3 month NIHSS or mRS). For this part of the analysis the dataset was split according to dichotomized revascularization success. In this step, we calculated correlations with the lesion loads as generated by manually segmented 90-day follow-up imaging and as generated both by thresholds and by FASTER on acute imaging in order to validate our hypotheses.

A result of having as many as 121 brain regions delineated by the atlas is that the lesion load data is relatively sparse. Our data thus fails to satisfy the normality assumptions of parametric statistical tests, leading to most of the correlations we observed being significant. To better observe the true significance of our findings, we used the non-parametric statistical method of bootstrap sampling (33) to construct confidence intervals (CI) for the obtained correlation values. One thousand samples were drawn from the original data (for every ROI, respectively) and individually correlated with the clinical outcome scores. The obtained correlations were then used to create a 95% confidence interval. If the confidence interval included zero correlation the original correlation was termed statistically insignificant and significant otherwise. Since we consider our study an exploratory one, examining the feasibility of linking lesion loads to NIHSS (rather than a study to identify which regions are linked to NIHSS), we do not perform any correction for multiple comparisons. As a result, this study should not be seen as identifying any individual stroke damage locations as “significantly” related to NIHSS, but rather as providing a group of regions which may, in a subsequent study of a larger group of patients, be viewed as good candidate regions on which to focus.

## Results

We analyzed 55 patients in total: the successful revascularization cohort contained 35 patients, whereas the unsuccessful revascularization cohort consisted of 18 patients. The successfully revascularized cohort entails 13 female and 23 male patients. The mean age of the group is 61 ± 12 years; minimum and maximum ages are 35 and 81, respectively. In the unsuccessfully revascularized cohort the mean age is 59 ± 14 years; minimum and maximum ages are 18 and 76, respectively. The group is composed of 7 female and 12 male patients.

### Lesion distribution

**Figure 3:**
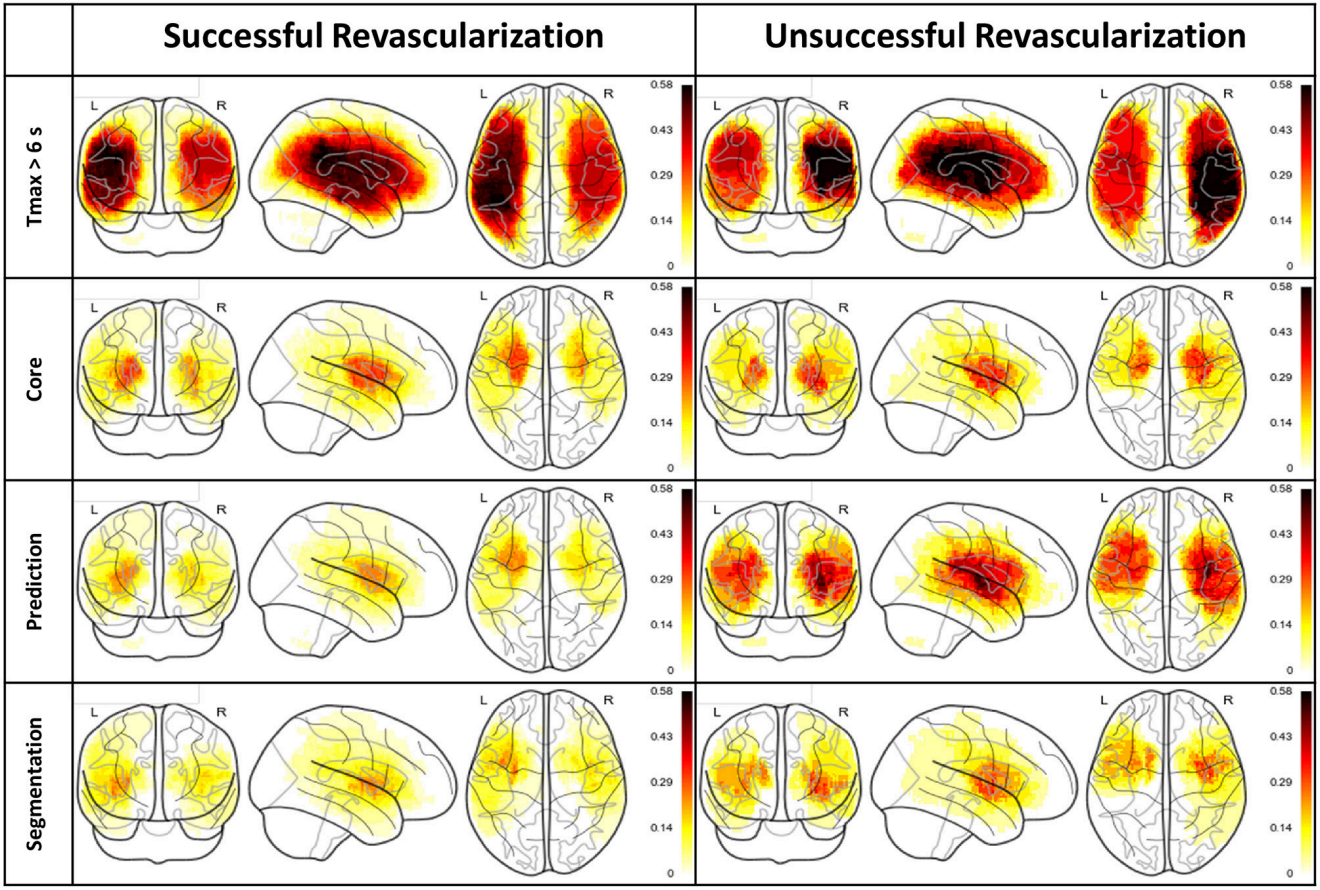
Lesion distributions horizontally and vertically grouped by revascularization outcome and type of lesion delineation, respectively. The distributions are normalized so that the values are confined to the range 0 (i.e., not affected by any lesion in the cohort) and 1 (i.e., affected by all lesions in the cohort).

Figure 3 depicts normalized lesion distributions grouped by underlying lesion delineation (rows) and revascularization outcome (columns).

### Correlations analysis

This section presents the results of the correlation between lesion loads and 3-month clinical assessments.

#### Three month NIHSS vs. mRS on complete dataset

**Figure 4:**
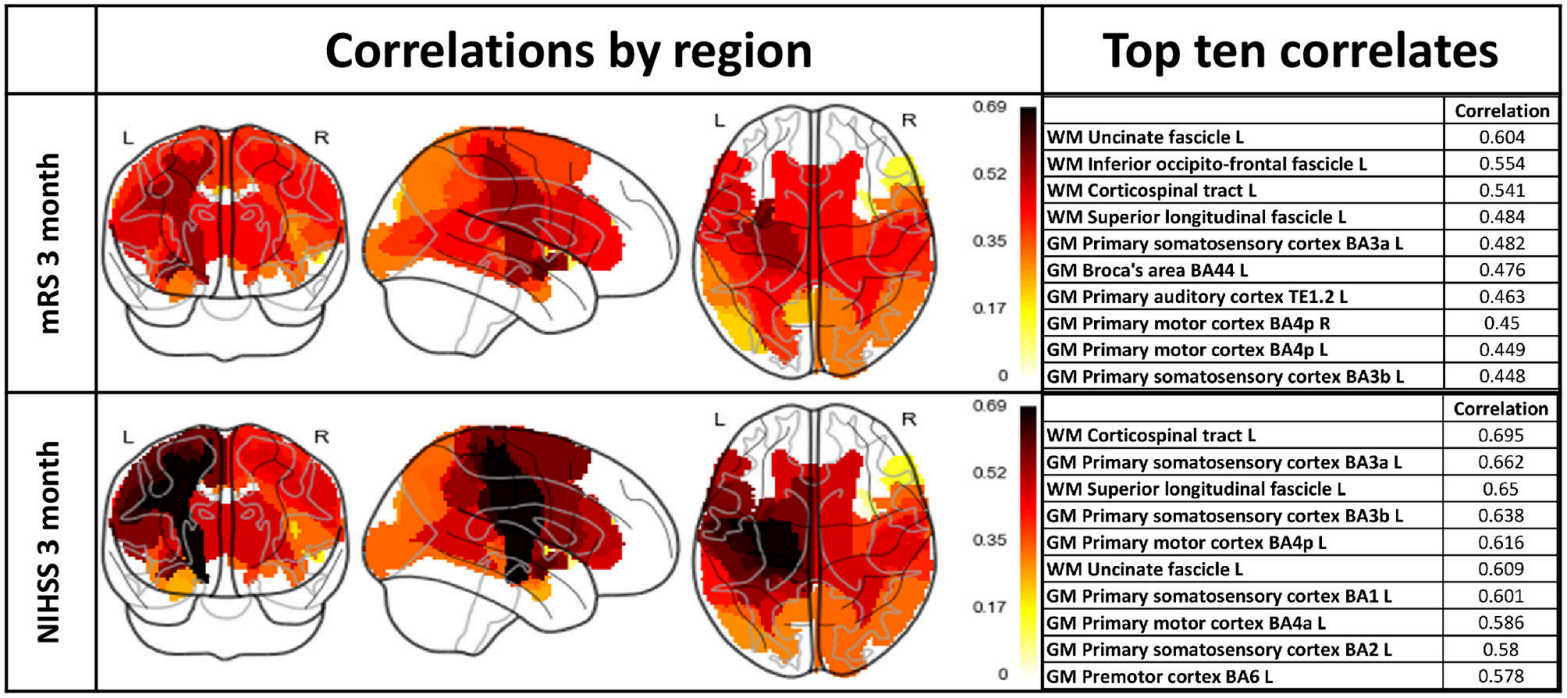
Lesion load correlations with 3 month mRS and NIHSS scores including the whole patient cohort. Left column: Visualization of regional lesion load correlations with clinical assessments. Right column: Top ten correlating regions with respect to outcome scores.

The results are depicted in Figure 4 both visually and in tabular form (only top ten regions).

A Wilcoxon signed-rank test comparing NIHSS and mRS correlations revealed a statistically significant difference between the two samples (*p*-value < 0.05).

#### Comparison of lesion delineations

Since NIHSS was found to yield significantly higher correlations than mRS, we used NIHSS as a measure of clinical outcome in the remainder of our experiments.

**Figure 5:**
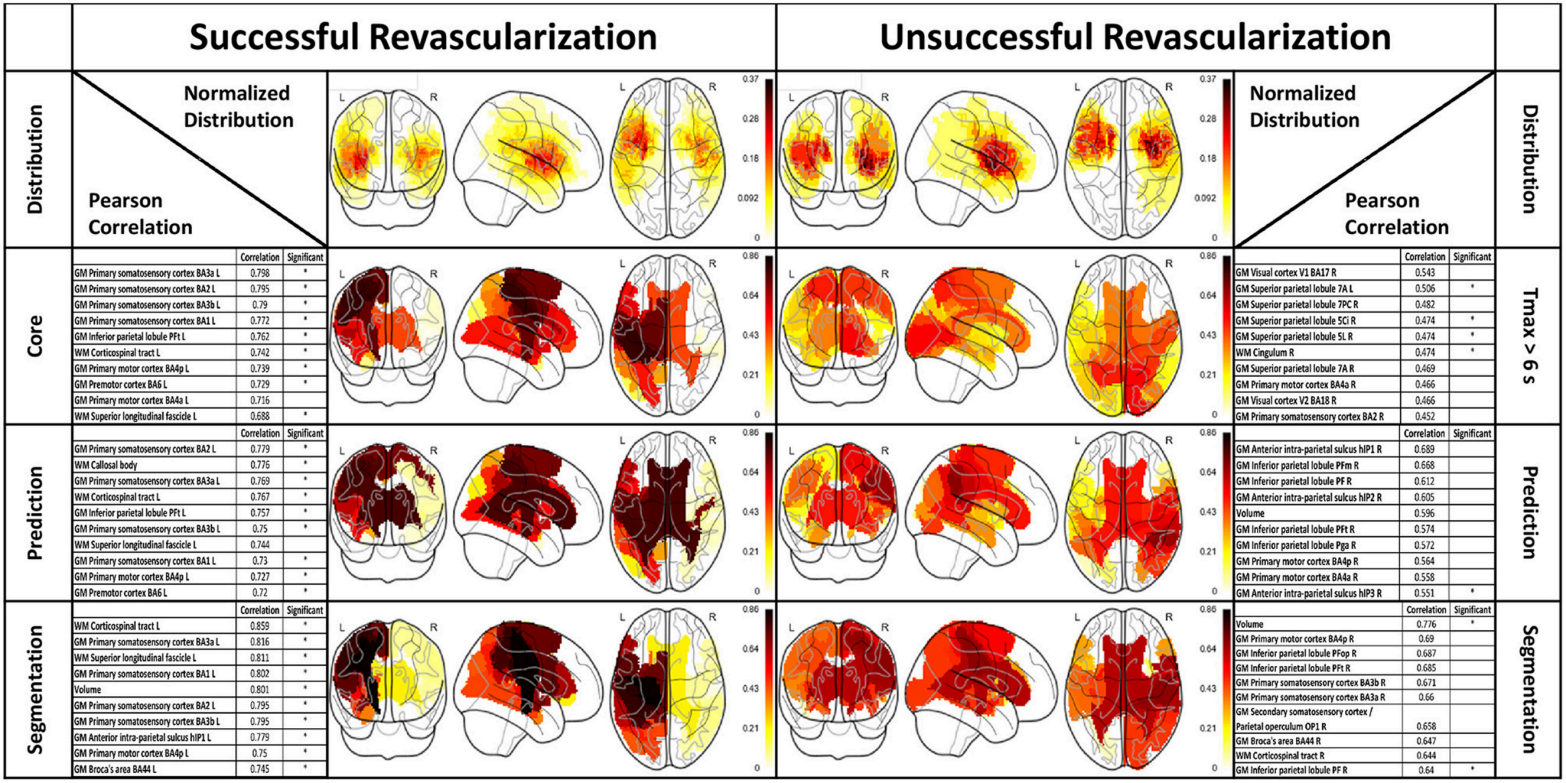
Correlation between 3 month NIHSS scores and structural ROIs (gray- and white-matter). The columns group the results according to revascularization outcome. Top row: Distribution of follow-up segmentations grouped by successful and unsuccessful revascularization. Rows 2–4: Correlation results for lesion loads based on different lesion delineations with top ten regions listed in tabular form. The tables include the total lesion volume correlations. Asterisks in the “Significant” column of the tables designate correlations that were found significant according to the bootstrap CI.

A correlation analysis was performed with respect to the 3 month NIHSS on the split dataset. The correlations are shown as overlay to a glass brain and in tabular form in Figure 5. The first row depicts the normalized lesion distributions for successful and unsuccessful revascularization based on the follow-up segmentation, whereas the subsequent rows with the respective tables present correlations. Lesion distributions (already presented in Figure 3) are shown only as a guide for interpretation. Correlations that were found significant according to the bootstrap CI are marked with an asterisk in the designated column of the table.

## Discussion

We proposed a methodology suitable for investigating a hypothesized relationship between affected functional brain regions and 3 month clinical outcome in ischemic stroke patients. Other studies have examined such a relationship on a voxel based level—i.e., voxel-based lesion symptom mapping (VLSM) for multiple sclerosis lesions (34). Another study found, through VLSM, a specific motor pathway influence on mRS outcome and a reflection of lateralized functions such as neglect and aphasia (35). Although the study included 101 patients it was nevertheless designated to be exploratory; this is a result of two limitations associated with VLSM: generally a large number of voxels must be considered and there is functional cross-dependence between the voxels. These two limitations can be alleviated with a regional approach, where voxels are grouped into functionally meaningful regions.

We analyzed the lesion distributions of 55 stroke patients, of which 35 were successfully revascularized (TICI 2b-3) and 18 were unsuccessfully revascularized (TICI 0-2a). As expected, the outcome prediction maps from FASTER show strong dissimilarities between successfully revascularized patients (where the prediction is based on a model trained on patients with TICI 3) and unsuccessfully revascularized patients (where the prediction is based on a model trained on patients with TICI 0). The segmentation distribution displays the same disparity between treatment outcome cohorts: this effect is less pronounced, which can perhaps be explained by the effect of collateral circulation preserving tissue which might otherwise have been lost, or the effects of partial revascularization in patients with a TICI greater than zero.

Our results show substantial differences in the revascularized and unrevascularized cohorts, with respect to correlations between lesion load and functional outcome, which is to be expected. In the case of successful revascularization, the tissue damage is mostly limited to tissue lying in the ischemic core, which is well-identified by diffusion-weighted imaging. This can be observed in the similarities between the loads observed in the ischemic core, FASTER prediction and final outcome segmentation. In this cohort, regions in the sensorimotor system were strongly correlated with outcome, as were the three white-matter tracts (superior longitudinal fascicle, corticospinal tract, and callosal body) as well as the total lesion volume. For the unrevascularized cohort, the situation is less well-defined. First, when considering the relationship between follow-up segmentation and NIHSS, only two of the top-ten correlated regions were significantly correlated according to the bootstrap confidence interval. The results arising from the Tmax and FASTER segmentations should be viewed in this light, since we should not expect assessments at the acute phase to be more accurate than the final lesion volume. Nevertheless, we can make a qualitative assessment of the most correlated regions: these differ not only in values but also in appearing regions. Correlations between prediction-based lesion loads and NIHSS were in general higher than between Tmax-based lesion-loads and NIHSS, but more correlations were found to be significant using Tmax than using prediction. We hypothesize that the former effect would persist in larger cohorts, and that the second would not, but this will require analysis of larger cohorts of unsuccessfully revascularized patients.

A major finding from our study is the difference in relationships between atlas based lesion load mapping and the different outcome measures (mRS and NIHSS). These scores have been developed to ensure simplicity, reliability and validity in its clinical application (36), and were tested for consistency (37) and reliability (38). The modified Rankin scale has been found to relate more closely to quality of life compared to the NIHSS (39). If, however, the focus is on specific neurological, rather than global disabilities, the NIHSS should be used (40). Our analysis revealed a statistically significant difference between the obtained correlation values, with correlations between lesion loads and NIHSS being higher than with mRS. A similarity in recognizable contours can be perceived, e.g., corticospinal tract and uncinate fascicle. However, different regions dominate and the heatmap as well as the tabular listing reveals that, in general, higher correlations were achieved with the NIHSS score. This might be an important finding for future investigations as recent studies focused on the mRS (35, 41). Moreover, white-matter structures dominate the top correlates with mRS. The NIHSS correlations also exhibit the importance of white-matter structures (50% white-matter regions in top six); the highest correlation was achieved by the left corticospinal tract. Besides the white-matter structures, both correlation lists exhibit the importance of sensorimotor structures. The importance of motor regions as well as white-matter structures with respect to outcome has been shown (35, 42). The observed correlation values together with the prominence of established brain areas are a strong support for the methodology of correlating lesion loads with NIHSS outcome assessment. The predominant white-matter tracts that appeared in our analysis were the corticospinal tract, cingulum, callosal body and superior longitudinal fascicle. The role of the corticospinal tract has been investigated and its importance with respect to stroke recovery outcome is established (14, 16, 43–47). This is consistent with the role of the corticospinal tract as the main pathway for afferent and efferent signal conduction between brain and limbs. The effect of an impact on the other white-matter structures is less clearly understood and can range from mental disorder (anterior cingulum) to working memory impairment (posterior cingulum) and loss of language integration ability (superior longitudinal fasciculus). Also, the dorsal Anterior Cingulate Cortex (dACC) was found to send task specific modulatory signals to the Supplementary Motor Area (48), which is an indication of its involvement in motor control.

Total acute lesion volume as measured through perfusion or DWI imaging has been found to be positively correlated with clinical assessments (12, 49, 50). Our results support these findings, but suggest that total lesion volume may not be the most important feature in predicting functional outcome: total lesion volume was surpassed by at least four gray-matter regions and/or white-matter structures in five out of the six analyzed situations (only in case of the unsuccessful segmentation result was the volume the top correlate). Total lesion volume for the various delineations ranked 1st (0.776, significant), 5th (0.801, significant), and 5th (0.596, unsignificant) for segmentation unsuccessful, segmentation successful and prediction unsuccessful, respectively. This suggests that while lesion volume is informative and carries predictive weight, it accounts only partially for the observed recovery. The importance of lesion location in predicting stroke outcome is supported in the literature, specifically in studies where the focus is on particular structures and brain regions (e.g., corticospinal tract) (14–17, 28, 43, 44, 46, 47). A study including loads on 132 cortical and subcortical regions constructed a decision tree associating regional lesion loads with outcome; the total lesion volume played a role in the left hemisphere, according to the study (41). Like our study, the number of patients precludes identifying loads in any one region as being significantly associated with outcome.

Our results confirm the significant and intertwined role of stroke lesion volume and location, and suggest that a number of other gray-matter regions and white-matter structures carry predictive information and may have the potential to increase the predictive accuracy.

A lateralization of the correlation values was evident in the results, with good correlations between left-hemispheric regions for successful thrombectomy and right-hemispheric regions for unsuccessful thrombectomy.

Studies have shown that the importance of regions with respect to outcome may depend on hemispheric idiosyncrasies: limbic, default mode and language areas in the left hemisphere, and visuospatial and motor regions in the right hemisphere (41). Areas of lateralized brain functions together with motor areas were found to influence functional outcome (35). However, we do not consider that our study has the statistical power to conclude that the observed effect is due to any biological reason, and assume that the lateralization is a result of the small patient cohort and, therefore, due to randomness in the lesion distribution with respect to laterality. This conclusion should be investigated and confirmed on a larger dataset. Similarly, the difference in statistical significance between unsuccessful and successful revascularized patient cohorts is likely a reflection of the sample sizes of the two cohorts, which was 18 and 35 for unsuccessful and successful groups, respectively. It is important to keep in mind the meaning of a 95% confidence interval derived from bootstrapping: by randomly sampling repeatedly from our available data, we simulate a large number of experiments. The confidence interval for the correlation coefficient is defined as the 2.5th and 97.5th percentiles of the derived correlations. Since the lesion loads were sparse, for many of these trials no patients with non-zero lesion load were randomly selected, and this accounts, in the main, for the large number of non-significant correlations. The results that the FASTER lesion predictions are superior to simple threshold-based delineations (in particular in case of unsuccessful revascularization) and that the predictive value of total lesion volume is surpassed by lesion loads must be confirmed in future investigations, ideally with substantially larger patient cohorts. We note that there may be brain regions not identified by this study which are nonetheless important for predicting stroke outcome. Regions which due to cohort size were not loaded in our dataset cannot be considered in our analysis: this again necessitates an increased cohort size. Additionally, lesion load on additional regions not represented in the Juelich atlas (e.g., the basal ganglia) may also influence clinical outcome. As a next step, atlases that encompass the deep brain nuclei, the inferior temporal lobe and frontal lobe areas should be included into the analysis.

The study investigated the relationship between lesion loads and clinical outcome assessment with the Pearson correlation coefficient. While we think the employed measure is applicable to the data, various alternative measures might be worth considering in future investigations.

Finally, correlations for each region were considered individually, while the effects of loads on networks of regions may not be adequately explained by loads on the constituent parts of those networks. Furthermore, adjacent regions will tend to be loaded together, and it is possible that loads on a given region may be predictive of clinical outcome, simply by their proximity to adjacent eloquent areas. Detailed NIHSS scores of the 15 functional elements and supplementary quality of life scales, providing functionality of subjects in daily life, were not available in the cohort under investigation.

### Outlook

Our analysis lays a solid foundation for exploring the relationship between neurological assessments (mRS and NIHSS) and lesion site and extent. This knowledge can be used to focus on and extract from imaging a selection of important features to make reliable predictions on neurological scores. The keywords here, we believe, are “focus” and “selection” as only information that is focused and manageable allows for a model that can be built from a feasible amount of patient data. Further, it brings the advantage of permitting a model with enough simplicity that the prediction process can be understood and further insights gained.

## Conclusion

This study investigated the relationship between imaging-based lesion loads and 3 month clinical assessments. We analyzed various lesion delineations used for the computation of the lesion loads. Regions known to be associated with stroke outcome were confirmed and new potentially informative areas suggested. The results support the clinical utilization of the automatically predicted volumes from FASTER over the simpler DWI and PWI lesion delineations.

## Author contributions

SH: Study design, data preparation and analysis, statistical analysis and writing of the manuscript; RW: Study design, concept, data interpretation, and writing of the manuscript. LH: Data preparation; JG: Manuscript discussion and review; PM: Manuscript discussion and review, BW: Data interpretation, manuscript discussion and review, SJ: Data preparation, manuscript discussion and review, MR: Study design, concept, data interpretation and writing of the manuscript, RM: Study design, concept, data interpretation, statistical analysis, and writing of the manuscript.

### Conflict of interest statement

The authors declare that the research was conducted in the absence of any commercial or financial relationships that could be construed as a potential conflict of interest.

## Abbreviations

NIHSS: National Institute of Health Stroke Scale
TICI: Thrombolysis in Cerebral Infarction Scale
ADC: Apparent Diffusion Coefficient.
